# Anatomical Study of the Lateral Cutaneous Branch of the Intercostal Nerves in the Intramuscular Portion

**DOI:** 10.7759/cureus.107665

**Published:** 2026-04-24

**Authors:** Guillaume Huttin, Romain Mari, Raymond Challita, Clémentine Rieussec, Gauthier Caillard, Pierre-Yves Rabattu, Alexandra Forli, Thibault Lafosse, Michael Bouyer

**Affiliations:** 1 Department of Reconstructive Surgery, Hand Surgery Unit, Centre Hospitalier Universitaire de Grenoble Alpes, Grenoble, FRA; 2 Department of Anatomy, Université Grenoble Alpes, Grenoble, FRA; 3 Department of Plastic and Reconstructive Surgery, Faculty of Medicine, Lebanese University, Beirut, LBN; 4 Department of Pediatric Surgery, Centre Hospitalier Universitaire de Grenoble Alpes, Grenoble, FRA; 5 Alps Surgery Institute, Hand, Upper Limb, Brachial Plexus, and Microsurgery Unit (PBMA), Clinique Générale d'Annecy, Annecy, FRA

**Keywords:** anatomical study, intercostal muscle, intercostal nerve, intercostal space, lateral cutaneous branch

## Abstract

Introduction: The lateral cutaneous branch (LCB) of the intercostal nerves (ICNs) is classically described as a sensory nerve supplying the lateral thoracic wall. However, it may also contribute to the innervation of the intercostal muscles and the digitations of the serratus anterior.

Objective: This study aimed to investigate the course, variations, and divisions of the LCB along its path within the intercostal space.

Methods: This anatomical study was conducted between January and April 2024 on 11 formalin-fixed adult cadavers. The third to sixth ICNs were dissected. The origin of the LCB relative to the midaxillary line, its length before division, and the number and destination of its branches (muscular or cutaneous) were recorded.

Results: Five females (45%) and six males (55%) were included (age range: 66-103 years). A total of 75 LCBs were identified and dissected. The mean distance from the midaxillary line was 3.72 cm ± 2.43 cm, and the mean length before division was 3.71 cm ± 1.84 cm. The LCB was divided into two branches in 56 cases (75%) and into three branches in 19 cases (25%). One branch consistently demonstrated a cutaneous distribution, while one or two branches exhibited a motor distribution.

Conclusion: Within its intramuscular course, the LCB provides motor branches to the intercostal muscles and the digitations of the serratus anterior before becoming purely sensory.

## Introduction

In their 1932 study, anatomists from King's College, England, precisely described the anatomy of the intercostal nerves, their branches of division, and their relationships with the muscular layers of the intercostal space [1]. Arising from the bifurcation of the thoracic spinal nerves after their emergence from the intervertebral foramen, the intercostal nerves constitute their anterior ramus. After entering the intercostal space at mid-height, the intercostal nerve approaches the upper rib of the space and then courses beneath its costal groove together with the intercostal artery, initially between the innermost and external intercostal muscles, and subsequently between the innermost and internal intercostal muscles. Within the intercostal space, near the midaxillary line, the nerve divides into three branches. A main branch continues its course within the same intercostal space, giving off multiple muscular branches along its trajectory and terminating at the anterior part of the thorax after passing through the internal intercostal and the pectoralis major muscles as a cutaneous branch known as the anterior cutaneous branch. A secondary branch of small caliber, rarely described, referred to as the "collateral branch," courses along the inferior aspect of the intercostal space, giving off communicating branches with the main branch of the subjacent intercostal space [2]. Finally, a branch, known as the lateral cutaneous branch (LCB), follows a short course along the intercostal muscles, first between the innermost and internal intercostal muscles and then between the internal and the external intercostal muscles. It pierces this outer layer near the midaxillary line to innervate the lateral aspect of the thorax by dividing into two branches (one anterior and one posterior) supplying the skin. Since this initial description, the LCB has been regarded as a purely sensory nerve.

Recent anatomical studies of the LCB have focused on its cutaneous course after its emergence from the intercostal space. This branch is of particular interest in breast reconstruction due to its contribution to the innervation of the mammary areola. However, we did not identify any study describing the intramuscular course of the LCB [3-5].

The contribution of the LCB to the muscular innervation of the intercostal muscles or the serratus anterior may have implications in nerve transfer surgery for brachial plexus palsy. In the rare cases where it is mentioned, this branch is used only for sensory-targeted transfers [6,7].

The aim of this study is to investigate the LCB within the intercostal space in order to examine its course, possible anatomical variations, and the presence of any potential motor innervation.

This article was previously presented as a meeting abstract at the 106e Congrès de l'AM (Associations des Morphologistes) on March 14, 2025.

## Materials and methods

A total of 11 formalin-fixed adult cadavers (five females and six males) from the Department of Anatomy, Histology, and Anthropology at Grenoble Alpes University, France, were dissected between January and April 2024. The study protocol was approved by our local ethics committee and registered under the number 24-2023.

Inclusion criteria were as follows: female or male subjects, with no restriction on age at the time of death, and no prior dissection of the thorax. Exclusion criteria were as follows: major thoracic cage malformation or deformity, and the presence of scars on the thorax overlying the dissection area or local thoracic conditions preventing dissection of the majority of the intercostal spaces relevant to the study. Due to unilateral thoracic alterations in one cadaver, an additional subject was included to achieve the planned sample size of 20 hemithoraces.

All dissections were performed by a single operator. The subjects were placed in the supine position with the arms abducted at 90°. When feasible, dissections were performed bilaterally. The intercostal nerves were exposed in the third to sixth intercostal spaces and then dissected from the midaxillary line to the sternum after detaching the lower head of the pectoralis major (extending from the sixth to the third rib). The LCB was identified from its origin and dissected along its intramuscular portion until its division. The length of the LCB (cm) and its origin relative to the midaxillary line (cm) were then measured. The number of branches and their destination (muscular or cutaneous) were recorded. Statistical analyses were performed using Excel 2016 (Microsoft® Corp., Redmond, WA). Means and standard deviations were systematically calculated. The correlation between two variables was assessed using linear regression along with the calculation of Spearman's correlation coefficient.

## Results

**Figure 1:**
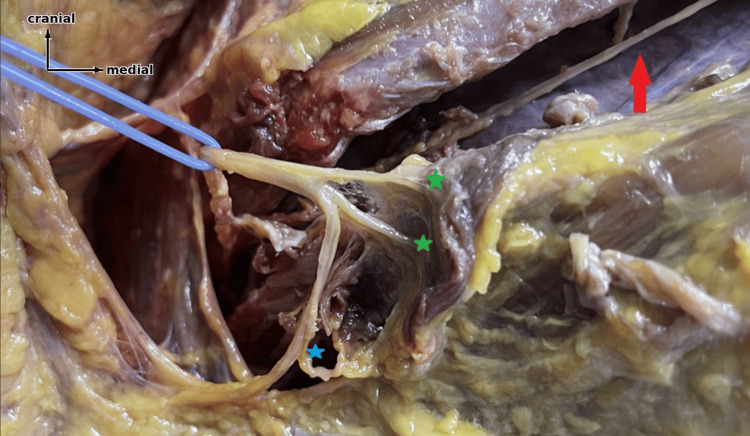
Cadaveric dissection of the LCB Red arrow: main branch; green stars: muscular branches; blue star: cutaneous branch; blue surgical loop: lateral cutaneous branch (LCB).

**Figure 2:**
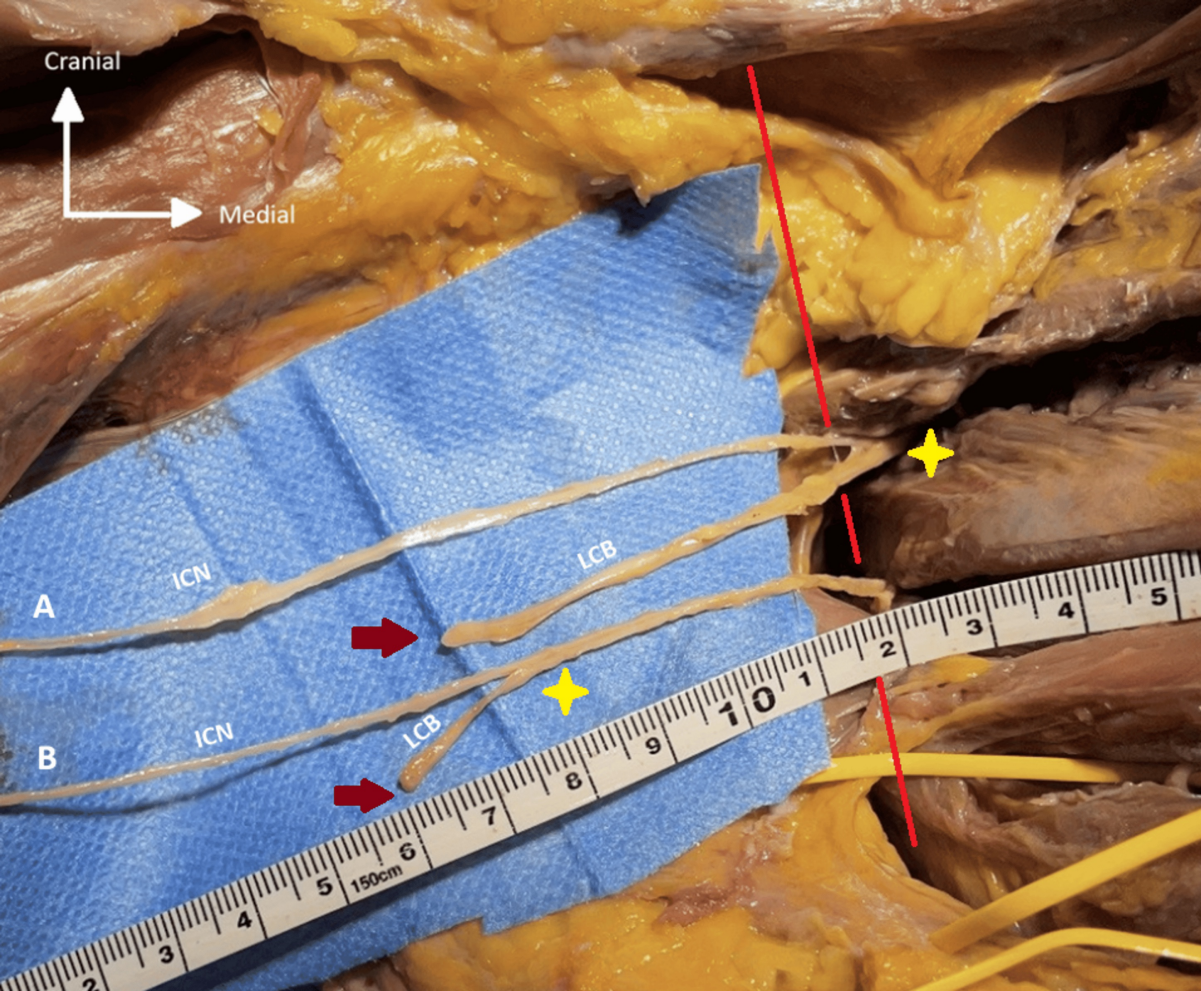
Photograph illustrating the relationship between the length of the LCB and its origin relative to the midaxillary line in two adjacent intercostal spaces The midaxillary line is shown in red, the branch origin is marked by a yellow star, and its division into multiple branches (here sectioned) is indicated by a dark red arrow. ICN: intercostal nerve; LCB: lateral cutaneous branch A: Long LCB with an origin close to the midaxillary line. B: Short LCB with an origin farther from the midaxillary line.

Data from the dissected subjects are summarized in Table 1. The LCB could be identified in 93.75% of the dissected intercostal spaces (75 cases). Its origin relative to the midaxillary line averaged 3.72 cm (± 2.43 cm). The branch followed a consistent course through the muscular layers, between the internal and external intercostal muscles, obliquely forward and downward. It was divided into two branches in 56 cases (75%) and three branches in 19 cases (25%), with equivalent diameters (Figure 1). Among its divisions, one branch had a cutaneous distribution, piercing the outer layer of the intercostal muscles to innervate the skin of the lateral thoracic wall. The remaining branches had a muscular distribution, supplying the external intercostal muscles and the digitations of the serratus anterior. The mean length of the LCB from its origin to its division was 3.71 cm (± 1.84 cm). The data relative to each intercostal space are summarized in Table 2. There was an inverse correlation between the origin of the LCB relative to the midaxillary line and the length of the LCB (rho = -0.45; p < 0.001). The longer the branch, the closer its origin was to this line (Figure 2).

**Table 1:** Demographic and dissection-related characteristics of the studied cadavers (n = 11)

Variable	Value
Sex	
Female	5 (45.5%)
Male	6 (54.5%)
Anthropometric variables	Mean ± SD
Age at death (years)	80.5 ± 11.1
Height (cm)	163.6 ± 10.5
Weight (kg)	65.9 ± 15.0
BMI (kg/m²)	24.5 ± 4.3
Dissection-related variables	
Mean interval between death and dissection (days)	59.5

**Table 2:** Measurements of the lateral cutaneous branch (LCB) for each intercostal nerves (ICN3-ICN6)

Variables	ICN3	ICN4	ICN5	ICN6
Lenght of the LCB (cm), mean ± SD	3.42 ± 1.51	3.54 ± 1.50	3.81 ± 2.48	4.19 ± 1.88
Origin of the LCB relative to midaxillary line (cm), mean ± SD	2.71 ± 2.22	3.73 ± 2.06	4.24 ± 2.60	4.34 ± 2.72

## Discussion

The LCB appears to be an anatomically consistent branch of the intercostal nerves, with a reproducible course and a component with motor distribution (one or two branches). Nevertheless, variability was observed in its size and origin.

Several authors have investigated the anatomy of the intercostobrachial nerve (ICBN), the LCB of the second intercostal nerve that communicates with the medial aspect of the arm. Van Tonder et al. reported in their study a motor component arising from this branch and supplying the pectoralis minor and pectoralis major muscles. However, this variation was observed in only two subjects and was unilateral in this study [8]. Koizumi et al. described the contribution of a branch termed the extramural nerve, arising from the LCB, which provides muscular innervation to the pectoralis major. This branch may correspond to the muscular branches described in our study, although the exact muscular innervation observed in our series differs [9]. An embryological study by Homma et al. also reported, during embryonic development, the presence of muscular branches originating from the LCB that supply the lateral thoracic wall [10]. A study by Nasu et al., investigating the innervation of the serratus anterior muscle, reported that the inferior digitations receive, in addition to the primary innervation from the long thoracic nerve, a muscular branch originating from the intercostal nerves. This branch could potentially correspond to the LCB. However, the anatomical description provided in the study lacks sufficient detail regarding its course and topographical relationships to allow for a definitive identification [11]. Taken together, these observations suggest that this cutaneous branch may indeed possess a muscular component.

This study has several limitations that should be acknowledged. Five intercostal spaces (6.25%) could not be studied due to local dissection difficulties related to old rib fractures (four cases) and the presence of a costal bone metastasis (one case). The advanced age of the population may explain the high rate of traumatic sequelae observed during the dissections, and the long preservation time before dissection (59.5 days) may have degraded the tissues and made the dissections more difficult. The use of formalin-fixed instead of fresh frozen cadaver may also have led to degradation of the soft tissue, by increasing their stiffness, potentially affecting the identification of fine neural branches and their precise relationships [12]. Only the third to sixth intercostal spaces were examined, which does not allow extrapolation of these findings to the entire thoracic wall.

While anatomists from King's College described a consistent emergence from the intercostal space of the LCB around the midaxillary line, recent anatomical studies contradict this observation, reporting variations in its point of emergence [2,4,13]. Our study supports this observation, showing considerable variation in the origin of the branch relative to the midaxillary line. However, the inverse proportional relationship observed between the length and the origin of the branch suggests a relative consistency in the position of its emergence after passing through the intercostal space.

Our team observed that intraoperative neurostimulation of the branch along its intramuscular course induces contraction of the external intercostal muscles. This finding led us to conduct the present anatomical dissection study, a necessary preliminary step for considering the LCB for nerve transfer aimed at restoring motor function. The anatomical findings of this study will need to be complemented by technical feasibility studies, as well as axonal counts to determine whether the LCB could have a role in the therapeutic arsenal of brachial plexus surgeons [14-16].

## Conclusions

This study shows that the LCB of the intercostal nerves, classically considered purely sensory, consistently contains a motor component along its intramuscular course. Its anatomy is reproducible, despite variability in its origin and length. Muscular branches to the external intercostal muscles and the serratus anterior challenge the traditional view of this branch as an exclusively cutaneous nerve.

These findings provide new anatomical insight and suggest that the LBC could be considered as a potential motor nerve donor for nerve transfer procedures, particularly in brachial plexus palsy surgery. Further studies with axonal quantification and surgical feasibility assessments are required to define its true clinical utility.

## References

[1] Davies F, Gladstone RJ, Stibbe EP (1932). The anatomy of the intercostal nerves. J Anat.

[2] Talsma J, Kusakavitch M, Lee D (2023). Forgotten branch of the intercostal nerve: implication for cryoablation nerve block for pectus excavatum repair. J Pediatr Surg.

[3] Smeele HP, Bijkerk E, van Kuijk SM, Lataster A, van der Hulst RR, Tuinder SM (2022). Innervation of the female breast and nipple: a systematic review and meta-analysis of anatomical dissection studies. Plast Reconstr Surg.

[4] Gardetto A, Hörmann R, Pfitscher K, Konschake M, Stofferin H (2024). Anatomical mapping of the 4th intercostal nerve's lateral cutaneous branch in both sexes: implications for advanced breast reconstruction. Surg Radiol Anat.

[5] Hwang K, Jung CY, Lee WJ, Chung IH (2004). The lateral cutaneous branch of the fourth intercostal nerve relating to transaxillary augmentation mammoplasty. Ann Plast Surg.

[6] Boulouednine M, Allieu Y (2001). Intercostal nerve transfer classification. Chir Main.

[7] Chuang DC, Yeh MC, Wei FC (1992). Intercostal nerve transfer of the musculocutaneous nerve in avulsed brachial plexus injuries: evaluation of 66 patients. J Hand Surg Am.

[8] van Tonder DJ, Lorke DE, Nyirenda T, Keough N (2022). An uncommon, unilateral motor variation of the intercostobrachial nerve. Morphologie.

[9] Koizumi M, Horiguchi M (1992). A study on the communication between the pectoral nerve and the extramural nerve branches of the intercostal nerves [Article in Japanese]. Kaibogaku Zasshi.

[10] Homma S, Shimada T, Wada I, Kumaki K, Sato N, Yaginuma H (2023). A three-component model of the spinal nerve ramification: bringing together the human gross anatomy and modern embryology. Front Neurosci.

[11] Nasu H, Yamaguchi K, Nimura A, Akita K (2012). An anatomic study of structure and innervation of the serratus anterior muscle. Surg Radiol Anat.

[12] Miyake S, Suenaga J, Miyazaki R (2020). Thiel's embalming method with additional intra-cerebral ventricular formalin injection (TEIF) for cadaver training of head and brain surgery. Anat Sci Int.

[13] Iida T, Narushima M, Yoshimatsu H (2013). Versatility of lateral cutaneous branches of intercostal vessels and nerves: anatomical study and clinical application. J Plast Reconstr Aesthet Surg.

[14] Costa AL, Papadopulos N, Porzionato A (2021). Studying nerve transfers: searching for a consensus in nerve axons count. J Plast Reconstr Aesthet Surg.

[15] Mioton LM, Dumanian GA, De la Garza M, Ko JH (2019). Histologic analysis of sensory and motor axons in branches of the human brachial plexus. Plast Reconstr Surg.

[16] Freilinger G, Holle J, Sulzgruber SC (1978). Distribution of motor and sensory fibers in the intercostal nerves. Significance in reconstructive surgery. Plast Reconstr Surg.

